# Enhanced Chlorophyll Degradation Triggers the Pod Degreening of “Golden Hook,” a Special Ecotype in Common Bean (*Phaseolus vulgaris* L.)

**DOI:** 10.3389/fgene.2020.570816

**Published:** 2020-10-06

**Authors:** Bo Hu, Jinlong Zhu, Hongyan Wu, Kun Xu, Hong Zhai, Ning Guo, Yi Gao, Jiayin Yang, Danhua Zhu, Zhengjun Xia

**Affiliations:** ^1^Key Laboratory of Soybean Molecular Design Breeding, Northeast Institute of Geography and Agroecology, Chinese Academy of Sciences, Harbin, China; ^2^University of Chinese Academy of Sciences, Beijing, China; ^3^Huaiyin Institute of Agricultural Science of Xuhuai Region, Huai’an, China; ^4^Zhejiang Academy of Agricultural Sciences, Hangzhou, China

**Keywords:** common bean (*Phaseolus vulgaris* L.), degreening, pod, transcriptome, cellulose

## Abstract

To reveal genetic factors or pathways involved in the pod degreening, we performed transcriptome and metabolome analyses using a yellow pod cultivar of the common bean “golden hook” ecotype and its green pod mutants yielded via gamma radiation. Transcriptional profiling showed that expression levels of red chlorophyll catabolite reductase (*RCCR*, *Phvul.008G280300*) involved in chlorophyll degradation was strongly enhanced at an early stage (2 cm long) in wild type but not in green pod mutants. The expression levels of genes involved in cellulose synthesis was inhibited by the pod degreening. Metabolomic profiling showed that the content of most flavonoid, flavones, and isoflavonoid was decreased during pod development, but the content of afzelechin, taxifolin, dihydrokaempferol, and cyanidin 3-*O*-rutinoside was remarkably increased in both wild type and green pod mutant. This study revealed that the pod degreening of the golden hook resulting from chlorophyll degradation could trigger changes in cellulose and flavonoids biosynthesis pathway, offering this cultivar a special color appearance and good flavor.

## Introduction

The common bean (*Phaseolus vulgaris* L.), as one of the most important legume crops worldwide, provides a major source of dietary protein, complex carbohydrates, dietary fiber, numerous vitamins, and trace minerals (Tharanathan and Mahadevamma, 2003). The common bean is cultivated from 52°N to 32°S latitude and from near sea level to elevations of more than 3,000 m. Among a large number of cultivated varieties and landraces, there is a high level of diversification in nutrient content, taste, color, and texture of pod and seed in the common bean.

Plant pigments, including carotenoid, anthocyanin, and chlorophyll, are in charge of fruit peel or flesh color (Tanaka et al., 2008). For a specific fruit or vegetable, the content and proportion of different pigments are the decisive factors for the color appearance. Chlorophyll is an essential photosynthetic pigment in chloroplast of higher plants and performs complex processes of harvesting light energy and driving electron transfer. Many fruit flesh or peel and leaves with green color are caused by the high chlorophyll content (Xu et al., 2020). However, yellowing or senescence is often accompanied by chlorophyll degradation. Chlorophyll and carotenoids are related to the color variation from green to yellow. When carotenoids are masked by excessive chlorophyll, the fruit flesh or peel and leaves appear green. The yellow color of carotenoids is unmasked upon chlorophyll degradation during ripening or senescence. Chlorophyll breakdown is a complicated multistep enzymatic process. *CLH* is a crucial factor in the regulation of the chlorophyll reduction in the pericarp (Harpaz-Saad et al., 2007). In two pear cultivars, one turned yellow during ripening due to loss of chlorophylls a/b and carotenoids, while the other stayed green until fully ripen, which could be accounted for the lower the expression levels of the genes for chlorophyll degradation, including *CLH*, *PAO*, and *NYC1*-like (Charoenchongsuk et al., 2015). Treatment with 1-MCP could maintain intact chloroplasts with well-organized grana thylakoids and small plastoglobuli and delay chlorophyll degradation by suppressing the expression of *PAO*, *NYC*, *NOL*, and *SGR1* (Cheng et al., 2012). The *SGR* gene and its homolog, *SGR*-like, had been detected in various plant species; overexpression of *SGRL* reduced the chlorophyll content and promoted chlorophyll breakdown (Sakuraba et al., 2014). Synthesis and degradation of chlorophyll are under control of the coordinate regulatory cascade, in which the malfunction of key steps or genes would affect total chlorophyll content and the color appearance.

The key factor that restricts the quality of fresh pods is the cellulose content. The tender pods of common beans with low cellulose content have a pleasant taste. In order to take full advantage of the common bean in human diet, it is necessary to improve the quality and reduce cellulose content. Cellulose biosynthesis is a complex biochemical process, which includes various enzymes, such as *CESA*, *Kor*, and *SuSy*. Uridine diphosphate-glucose (UDP-glucose) is regarded as the immediate substrate for cellulose polymerization in higher plants. Photosynthetically fixed CO_2_ is the ultimate source of C for the synthesis of nucleotide sugars, such as UDP-glucose, which are the building blocks for synthesis of cell wall polysaccharides (Nakai et al., 1999). UDP-glucose can be derived from the cleavage of sucrose catalyzed by *SuSy* yielding UDP-glucose and fructose, demonstrating that *SuSy* had tight association with cellulose synthesis and the availability of sucrose in the cell would affect the rate of cellulose synthesis (Coleman et al., 2009).

Flavonoids, including flavone, flavonol, flavanone, isoflavone, and anthocyanin, constitute an important group of plant secondary metabolite, which can enable plants to adjust to environmental pressures (Kovinich et al., 2014). Recent researches showed that these compounds have physiological functions such as antioxidant, bacteriostatic, and anti-inflammatory, which are beneficial to human health. Especially isoflavonoid is predominantly synthesized in legumes plants. Anthocyanin, a class of flavonoids, localized in vacuoles, provided a wide range of colors ranging from orange/red to violet/blue. The content and variety of anthocyanins are the primary determinants of color in many fruit peel and flesh or flowers; the family of MYB and WD40 transcription factors and *DFR* and *CHS* had significant regulatory function on anthocyanin synthesis (Wang et al., 2019; Zhuang et al., 2019).

Among abundant germplasms, including landraces, ecotypes, and cultivars, there is a wide range diversification in pod and seed coat colors and patterns in the common bean. By fine mapping of QTL, the genes controlling the color of the seed coat, pod, stem, and flower were mapped on chromosomes 1 and 3; UDP flavonoid glycosyl transferase (*UGT*) was identified as the candidate gene for black seed coat in Adzuki Bean (Li et al., 2017). Through an Andean intragene pool recombinant inbred line (RIL) population, 23 QTLs for 6 pod traits were detected, and 4 QTLs for pod color were identified (Yuste-Lisbona et al., 2014). The R2R3 MYB transcription factor *TcMYB113* regulated green/red pod color in cacao (Motamayor et al., 2013). The characterization of the genetic variability of three stay-green common bean cultivars indicated high initial chlorophyll *a* content and reduced chlorophyll degradation throughout senescence. In the common bean, the pod color, especially purple, is well documented; Lablab with purple pods contained the pigments of anthocyanins and flavonol. The expression patterns of *LpPAL*, *LpF3H*, *LpF30H*, *LpDFR*, *LpANS*, and *LpPAP1* were significantly induced in purple pods compared to the green ones (Cui et al., 2016). In purple kidney bean pod, *PvMYB1*, *PvMYB2* (*R2R3 MYB* transcription factors), and *PvTT8-1* [basic helix–loop–helix (*bHLH*) transcription factors] might play a crucial role in transcriptional activation of most anthocyanin biosynthetic genes (Hu et al., 2015). A locus, *Prp* (purple pod), having five alleles affecting anthocyanin pigmentation of corolla and pod, was detailed in *Phaseolus vulgaris* L. The allele *Prp* produced dark purple corolla (Okonkwo and Clayberg, 1984). Chlorophyll degradation and chloroplast breakdown may be involved in degreening and be the foundation for the appearance of other colors; however, the regulatory mechanism at the molecular level has not been well studied in the common bean.

The construction of mutant libraries provides researchers and breeders with good resources for fundamental research or breeding materials. Dalong 1, having unique yellow and tender pods, is an important common bean ecotype with superior nutrition and is a favorite vegetable in northeast China. This cultivar with a determined growth habit does not require trellises to climb. We constructed a gamma radiation mutant library of Dalong 1. Among various phenotypic changes such as leaf morphology, pod dimension, and sterility, a higher proportion of green pod mutations were noted. In order to reveal the physiology and molecules mechanism underlying the yellow to green mutation, we first investigated the chlorophyll and cellulose content and performed transcriptome and metabolome analyses at different pod developmental stages. Key genes or metabolites triggered by the degreening of pod were revealed, which would provide new insights into the detailed regulatory mechanism of degreening process in the common bean.

## Materials and Methods

### Common Bean Ecotype of Golden Hook and Its Green Pod Mutation Lines

Common bean mutant population was constructed by exposing the seeds of cultivar Dalong 1 of ecotype of the golden hook to ^60^Co γ radiations at a dosage of 200 Gy. Phenotype observation was performed at M_3_ generation. A total of 76 mutant lines with green pods were noted in this mutant library. The wild-type Dalong 1 (yellow pod, YP) and the green pod mutant M628 (green pod, GP) were cultivated in Harbin City, Heilongjiang Province of China in 2018. After fertilization, the pods were sampled at different growth stages based on pod length: 2, 5, and 10 cm. In total, six treatments, e.g., YP-2, YP-5, YP-10, GP-2, GP-5, and GP-10, were sampled in triplicate. For further confirmation, we selected another two green pod mutant lines (M693 and M756) and one mutant line (M729) whose pod color was segregated (yellow pod named M729Y and green pod named M729G). Three biological replicates were collected per treatment. The samples were immediately frozen in liquid nitrogen and then stored at −80°C for the following experiments.

### Chlorophyll Content Measurement and Ultrastructure of Chloroplast

#### Chlorophyll Determination

Two hundred milligrams of plant sample was extracted in 25 ml of 80% (v/v) ethyl alcohol in a dark at room temperature for 48 h. The absorbance (A) of the supernatant was measured at 665, 649, and 470 nm, recorded as A665, A649, and A470, respectively. The pigment concentration was calculated using the following formulas:

$$\begin{array}{c} \text{Ca}(\text{mg}/\text{L})=13.95\text{A}665-6.88A649\\ \text{Cb}(\text{mg}/\text{L})=24.96\text{A}649-7.32A665\\ \text{Ct}(\text{mg}/\text{L})=\text{Ca}+\text{Cb}\\ \text{Cxc}(\text{mg}/\text{L})=(1000\text{A}470-2.05\text{Ca}-114.8\text{Cb})/245 \end{array}$$

The pigment content was calculated as pigment concentration multiplied by extraction liquid product and divided by fresh weight.

#### Chloroplast Ultrastructure

The pod tissues, 2 mm^3^ in volume, were fixed in 2.5% glutaraldehyde fixation solution at 4°C for more than 2 h. The tissues were washed with phosphate-buffered saline (PBS) at 4°C for 15 min, followed by washing with acetone of different concentrations (50, 70, 90, and 100%). After washing, the resin was soaked overnight and polymerized at a high temperature, sliced by a microtome (Leica, Germany), and was stained by uranium acetate and lead citrate. The copper mesh was covered on the sample drop for adsorbing for 5 min and stained with phosphotungstic acid for 30 s. The samples were examined with a transmission electron microscope (H-7650, Hitachi, Japan).

### Determination of Cellulose Content

#### Cell Wall Extraction

Sample (0.3 g) was added with 1 mL 80% ethanol, rapidly homogenized at room temperature, and then incubated at 90°C in a water bath for 20 min. After cooling, samples were centrifuged at 6,000 × *g* for 10 min, and the supernatant was discarded. The precipitate was washed using 1.5 ml 80% ethanol and acetone, respectively. One milliliter reagent I was added for soaking for 15 h to remove starch. After being centrifuged at 6,000 × *g* for 10 min, the supernatant was discarded; the precipitate was dried, weighed, and regarded as the cell wall mass (CWM).

#### Extraction of Cellulose

Five milligrams of dried CWM was homogenized in 0.5 ml distilled water. Then, 0.75 ml of concentrated sulfuric acid was slowly added to tubes in an ice water bath, mixed well and then incubated in ice for 30 min. The tubes were centrifuged at 8,000 × *g* at 4°C for 10 min; the supernatants were diluted with distilled water 20 times.

#### Cellulose Determination

One hundred fifty microliters of diluted supernatant was mixed with 35 μl working solution and 315 μl sulfuric acid. A blank tube was added with 150 μl distilled water, 35 μl working solution, and 315 μl sulfuric acid. After mixing, it was placed in water bath at 95°C for 10 min and cooled to room temperature. The absorbance values of blank and sample tubes were read at 620 nm: ΔA = A (sample tube) − A (blank tube). W stands for the dry weight of the cell wall mass.

Cellulose⁢content⁢(mg/g⁢dry⁢weight)=3.17×(Δ⁢A+0.0043)/W.

### RNA Extraction, cDNA Library Construction, and RNA Sequencing

Eighteen samples were used for the transcriptome analysis. Total RNA was extracted from three biological replicates of each sample using TRIzol reagent, according to the manufacturer’s instructions. RNA purity was checked using a NanoPhotometer spectrophotometer (IMPLEN, CA, United States). RNA concentration was measured using a Qubit RNA Assay Kit in Qubit 2.0 Flurometer (Life Technologies, CA, United States). RNA integrity was assessed using the RNA Nano 6000 Assay Kit of the Agilent Bioanalyzer 2100 system (Agilent Technologies, CA, United States).

A total of 1.5 μg RNA per sample was used as input material for the RNA sample preparations. Sequencing libraries were generated using NEBNext Ultra RNA Library Prep Kit (New England Biolabs, United States), and index codes were added to attribute sequences to each sample. The library preparations were sequenced on Illumina Hiseq platform, and paired-end reads were generated. Gene function annotation and DEGs were analyzed.

Gene function was annotated using the following databases: NCBI non-redundant protein sequences (Nr), Clusters of Orthologous Groups of protein (KOG), GO, and KEGG. All the unigenes were searched against KO, KOG, and KEGG databases using the BLASTX algorithm.

The clean reads were mapped back onto the assembled reference transcriptome (EnsemblPlants^1^), and gene expression levels were estimated by RSEM (Li and Dewey, 2011) for each sample. Differential expression analysis of two conditions/groups was performed using the DESeq R package. DESeq provides statistical routines for determining differential expression in digital gene expression data using a model based on the negative binomial distribution. The resulting P values were adjusted using the Benjamini and Hochberg’s approach for controlling the false discovery rate. Genes with an adjusted *P* < 0.05 found by DESeq were assigned as differentially expressed. For pathway enrichment analysis, all DEGs of each comparison group were mapped to pathways in the KEGG database by KOBAS software to identify significantly enriched KEGG pathways (Kanehisa et al., 2008).

### Real-Time Quantitative PCR Validation

For RT-qPCR, oligonucleotide primers were designed according to each gene’s transcript sequence with Primer 3 and Beacon Designer 7 software. *Actin11* (*Phvul.008G011000*) was used as the reference gene (Borges et al., 2012). RT-qPCR was carried out using SYBR Green-based PCR assay in LightCycler 96 (Roche, Switzerland). Each reaction mix contained 1.0 μl of complementary DNAs (cDNAs), 7.5 μl SYBR Premix ExTaq, 0.3 μl PCR forward primer (10 μmol L^–1^), 0.3 μl PCR reverse primer (10 μmol L^–1^), and 5.9 μl ddH_2_O, to a final volume of 15 μl. The PCR conditions were 95°C for 30 s, followed by 40 cycles of 95°C for 10 s, and 60°C for 30 s. The melting curve conditions were 95°C for 15 s, 60°C for 60 s, and 95°C for 1 s. Each RT-qPCR analysis was performed in triplicate, and the mean was used for RT-qPCR analysis. The relative expression of the genes was calculated according to the method of 2^–△△Ct^, and SPSS was used to analyze the data.

### Preparation of Metabolome Samples and Measurement by HPLC

The freeze-dried pod was homogenized using a mixer mill (MM 400, Retsch) with a zirconia bead for 1.5 min at 30 Hz. One hundred milligrams powder of pod was extracted overnight at 4°C with 1.0 ml 70% aqueous methanol. Following centrifugation at 10,000 × *g* for 10 min, the extracts were absorbed (CNWBOND Carbon-GCB SPE Cartridge, 250 mg, 3 mL; ANPEL, Shanghai, China^2^) and filtrated (SCAA-104, 0.22 μm pore size; ANPEL, Shanghai, China^3^) before liquid chromatography–mass spectrometry (LC-MS) analysis.

#### HPLC Conditions

The sample extracts were analyzed using an LC-electrospray ionization (ESI)-MS/MS system (HPLC, Shim-pack UFLC SHIMADZU CBM30A system^4^ ; MS, Applied Biosystems 6500 Q TRAP^5^). The analytical conditions were as follows: HPLC, column, Waters ACQUITY UPLC HSS T3 C18 (1.8 μm, 2.1 mm × 100 mm); solvent system, water (0.04% acetic acid):acetonitrile (0.04% acetic acid); gradient program, 100:0 V/V at 0 min, 5:95 V/V at 11.0 min, 5:95 V/V at 12.0 min, 95:5 V/V at 12.0 min, 95:5 V/V at 15.0 min; flowrate, 0.40 mL/min; temperature, 40°C; and injection volume, 2 μl. The effluent was alternatively connected to an ESI-triple quadrupole-linear ion trap (QTRAP)-MS.

#### ESI-Q TRAP-MS/MS

Linear ion trap (LIT) and triple quadrupole (QQQ) scans were acquired on a QTRAP mass spectrometer, API 6500 QTRAP LC/MS/MS System, equipped with an ESI turbo ion-spray interface, operating in a positive ion mode and controlled by Analyst 1.6 software (AB Sciex). The ESI source operation parameters were as follows: ion source, turbo spray; source temperature, 500°C; ion spray voltage (IS), 5,500 V; ion source, gas I (GSI), gas II (GSII), and curtain gas (CUR) were set at 55, 60, and 25.0 psi, respectively; the collision gas (CAD) was high. Instrument tuning and mass calibration were performed with 10 and 100 μmol/L polypropylene glycol solutions in QQQ and LIT modes, respectively. QQQ scans were acquired as multiple reaction monitoring (MRM) experiments with collision gas (nitrogen) set to 5 psi. DP and CE for individual MRM transitions were done with further DP and CE optimization. A specific set of MRM transitions was monitored for each period according to the metabolites eluted within this period.

### Statistical Analysis

Each experiment was repeated three times. All data were recorded as the means ± standard deviation (SD) and analyzed using a one-way analysis of variance (ANOVA) with SPSS software 19.0. Statistical difference between samples (^∗^*P* ≤ 0.05 was considered to be significant; ^∗∗^*P* ≤ 0.01 was considered to be extremely significant) was determined by Duncan’s test.

## Results

### A High Rate of Yellow-to-Green Mutation Was Observed in a Gamma Radiation-Induced Mutant Library

A mutant library was constructed by exposing seeds of Dalong 1 to ^60^Co γ radiations at a dosage of 200 Gy. Compared with the wild type, this mutant library displayed a wide range of phenotypic variations in traits, e.g., 100—seed weight, flower time, plant height, sterility, pod width, pod, and seed coat color among 3,500 plants in M_3_ lines. In contrast to yellow pod of wild-type Dalong 1, there were 76 mutant lines showing green pods or green and yellow segregation in a total of 607 M_3_ lines, much higher mutation rate than other traits such as sterility (33 mutant lines). Among the green pod mutant lines, the pod color was unanimously green; however, the green and yellow segregation displayed green and yellow pods segregation, approximately in a green to yellow ratio of 3:1 or 15:1.

**FIGURE 1:**
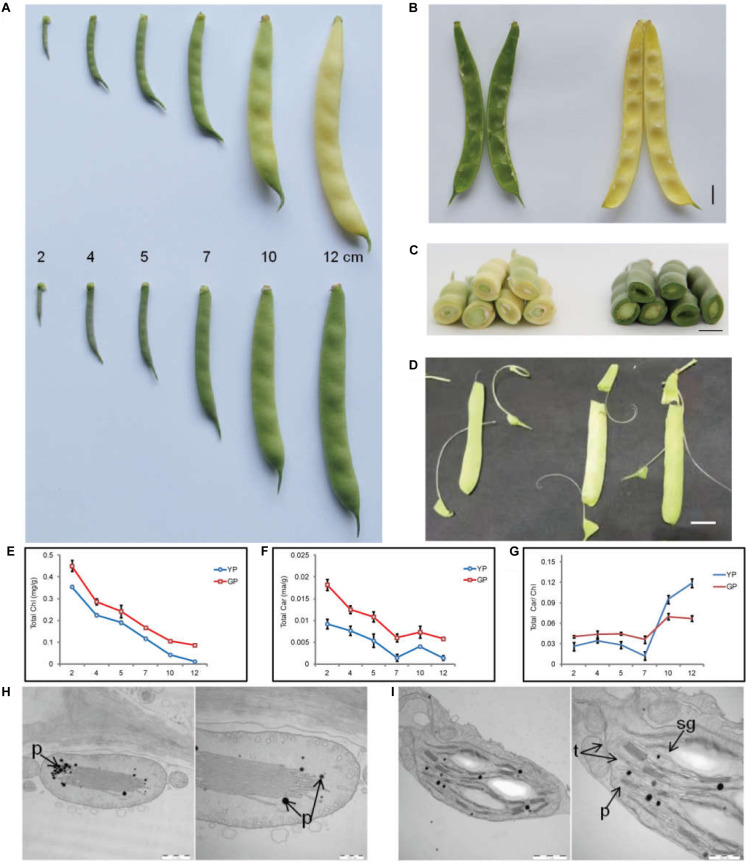
Pod color conversion, pigment content changes, and chloroplast microstructure during pod development. **(A)** Phenotypes of common bean pods of wild type (YP) and green pod mutant (GP). **(B,C)** phenotypes of pod interior and cross-section of the wild type and green pod mutant. **(D)** Phenotypes of green pod mutant with high cellulose content in the pods. **(E–G)** Total chlorophyll and carotenoid contents and the Car/Chl ratio at all developmental stages. **(H,I)** Structure of chloroplasts from wild type **(H)** and mutant **(I)**; t, thylakoids, sg, starch grain, p, plastoglobuli. Scale bar: 1 μm and 500 nm.

The pod color of wild-type Dalong 1 was green at the beginning of pod development (2 cm long), which rapidly turned to yellow as pod extended to approximately 10 cm long. The pod color of green pod mutant lines stayed green until eventual maturation. The pods of Dalong 1 appeared a little crooked, but green pod mutant was relatively straight (Figure 1A). From the intersection, the tissues underneath the epidermis of the pod also appeared green in consistent with pod appearance (Figures 1B,C).

Initially, a rapid colorimetric assay was employed to measure the chlorophyll and carotenoid contents during pod development. As early as the developmental stage of 2-cm-long pods, the chlorophyll and carotenoid contents in the green pod mutants were higher than that in the wild type. During pod development, the chlorophyll content in the wild type decreased sharply. In contrast, the chlorophyll content in green pod mutant lines only slightly decreased (Figures 1E–G).

The chloroplast ultrastructure of pods at 10 cm long in green pod mutant and wild type was compared using TEM. In contrast to the wild type, the chloroplast of the mutant remained a well-organized grana thylakoids and had more starch grain and less plastoglobuli (Figures 1H,I), indicating that the chloroplasts were capable of making chlorophyll.

### Transcriptome Profiles

**FIGURE 2:**
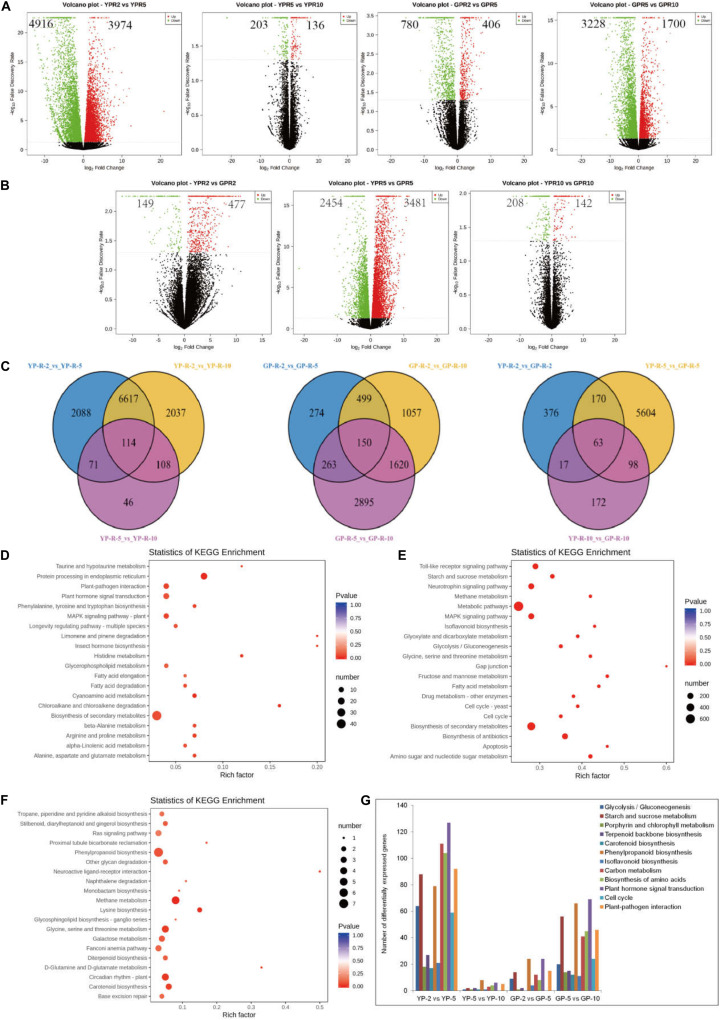
The differential expression genes analysis between wild type and green pod mutant by transcriptome. **(A,B)** The volcano plot showing the number of differential expression genes between two comparative groups. **(C)** Venn diagram showing common and specific DEGs numbers from different combinations displayed in the overlapping and non-overlapping regions, respectively. **(D–F)** Top 20 significantly enriched KEGG pathways in **(D)** YP-2 vs. GP-2, **(E)** YP-5 vs. GP-5, and **(F)** YP-10 vs. GP-10. **(G)** The statistics of DEGs in some metabolic pathway at two developmental stages of wild type and green pod mutant.

In order to reveal the transcriptional abundance of genes involved in degreening process, transcriptome was performed using equal amounts of RNA from pods. After removing low-quality reads, adaptor sequences, and ribosomal RNA (rRNA) reads, we obtained 50407645 (YP-2), 48995896 (YP-5), and 54170305 (YP-10) and 59445277 (GP-2), 51527246 (GP-5), and 49269434 (GP-10) clean reads, respectively (Supplementary Table S1). Clean reads were then mapped to the common bean reference genome using HISTAT software. Comparing YP-2 to YP-5, there were 4,916 genes downregulated and 3,974 genes upregulated, while only 203 genes were downregulated and 136 genes upregulated when YP-5 was compared with YP-10. In the green pod mutant, the down- and upregulated genes were 780 and 406 for GP-2 vs. GP-5 and 3,228 and 1,700 for GP-5 vs. GP-10 (Figure 2A). Three compared combinations (YP-2 vs. YP-5, YP-5 vs. YP-10, and YP-2 vs. YP-10 and GP-2 vs. GP-5, GP-5 vs. GP-10, and GP-2 vs. GP-10) shared 114 and 150 DEGs, respectively (Figure 2C).

To further determine the function of the DEGs during pod development, KEGG analysis was performed. KEGG enrichment analysis revealed that limonene and pinene degradation, histidine metabolism, fatty acid elongation, and protein processing in the endoplasmic reticulum were the prominently enriched for YP-2 vs. GP-2 (Figure 2D). Comparing YP-5 to GP-5, many pathways showed enrichment, such as fructose and mannose metabolism, fatty acid metabolism, amino sugar, and nucleotide sugar metabolism. Starch and sucrose metabolism and isoflavonoid biosynthesis were also noticeably enriched (Figure 2E). Neuroactive ligand–receptor interaction and D-glutamine and D-glutamate metabolism were the two most obvious enrichment pathways for YP-10 vs. GP-10; other pathways, e.g., circadian rhythm plant, phenylpropanoid biosynthesis, and carotenoid biosynthesis, were also enriched to a certain extent (Figure 2F).

As the pods extended, many marked changes occurred in numerous crucial pathways, such as glycolysis, starch and sucrose metabolism, porphyrin and chlorophyll metabolism, carotenoid biosynthesis, phenylpropanoid biosynthesis, carbon metabolism, and plant hormone signal transduction. In the wild type, radical changes in expression levels occurred from YP-2 to YP-5, while in the green pod mutation line, contrasting changes in expression levels were observed from GP-5 to Gp-10. Numerous pathways were activated at the early stages in the wild type; there were a large number of DEGs for YP-2 vs. YP-5 but fewer DEGs for YP-5 vs. YP-10. In contrast, more DEGs for GP-5 vs. GP-10 than GP-2 vs. GP-5 were detected (Figure 2G).

### Key Genes Involved in Chlorophyll Synthesis and Degradation Were Revealed by Transcriptome

As the chlorophyll content decreased with the pods degreening (Figure 1E), we focused on porphyrin and chlorophyll metabolism pathway. It was evident from Supplementary the figure that the expression levels of the genes for chlorophyll synthesis generally showed a downward trend during pod development both in wild type and green pod mutant (Supplementary Figure S3). The genes for chlorophyll synthesis, e.g., as *HEMC* (*Phvul.002G034500*), glutamyl transfer RNA (tRNA) reductase (*HEMA*, *Phvul.002G216100*), *CHLI* (*Phvul.003G057600*, *Phvul.006G178400*), and *POR* (*Phvul.005G083700*, *Phvul.011G148900*), were decreasing throughout in the wild type (Supplementary Figure S3A), whereas the genes for chlorophyll synthesis in the green pod mutant increased slightly from GP-2 to GP-5 and thereafter decreased (Supplementary Figure S3A). In particular, the transcription of *POR* (*Phvul.005G083700*) showed the opposite pattern when the pods extended from 2 to 5 cm, which was increased in the green pod mutant but decreased steeply in wild type (Supplementary Figure S3A).

**FIGURE 3:**
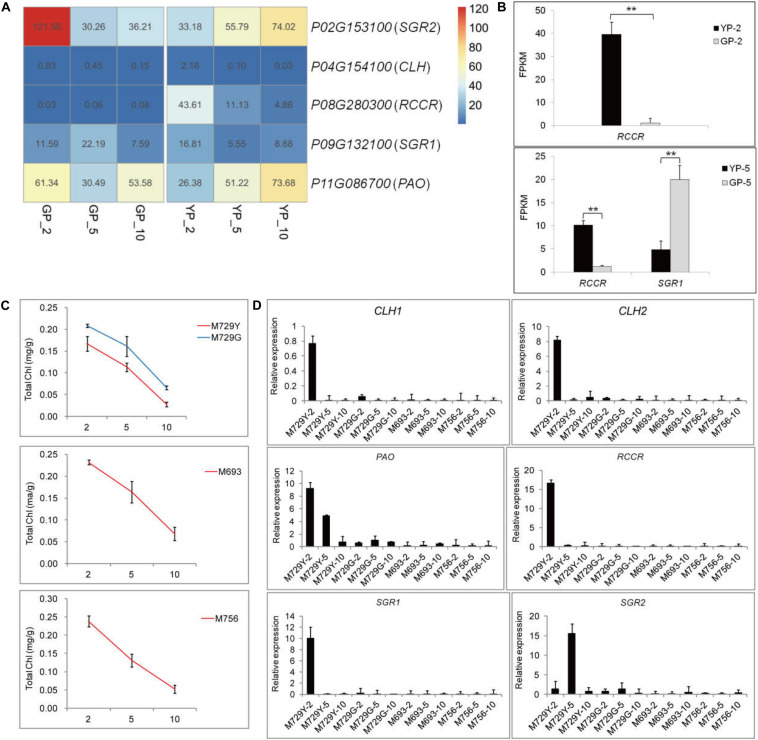
The differential expression genes analysis in porphyrin and chlorophyll metabolism pathway of YP, GP, and the other green pod mutant lines. **(A)** The changes in gene expression level of degradation in YP and GP, respectively. **(B)** Expression levels of DEGs for chlorophyll degradation between YP-2 and GP-2 and YP-5 and GP-5, respectively. **(C)** The chlorophyll content in yellow pods (M729Y) and three green pod mutants (M729G, M693, and M756), respectively. **(D)** Expression levels of DEGs for chlorophyll degradation successively in the M729Y, M729G, M693, and M756 mutant lines. Error bars represent the standard deviation of three replicates. Data were analyzed using the *t*-test. *p < 0.05, **p < 0.01.

The expression levels of chlorophyll degradation-related genes started to accumulate at early stage of wild type; notably, *RCCR* (*Phvul.008G280300*) had high expression levels at YP-2 (Figures 3A,B) and then gradually decreased. Similar trends were observed for the genes of *PAO* (*Phvul.011G086700*) and *SGR2* (*Phvul.002G153100*) in the wild type (Figure 3A). In the green pod mutant, the expression levels of *CLH* (*Phvul.007G278100*) increased gradually, and *SGR* (*Phvul.009G132100*) increased from GP-2 to GP-5 and then decreased sharply (Figure 3A). The expression levels of *RCCR* (*Phvul.008G280300*) in GP were extremely low throughout the pod development (Figures 3A,B). The aggravated degradation of chlorophyll together with the decreased synthesis resulted in the chlorophyll content in the pods decreased sharply, leading to the degreening phenotype.

The other four mutant lines (M729Y, M729G, M693, and M756) were also selected to analyze the variations of chlorophyll content and expression levels of genes involved in chlorophyll metabolism pathway. Similarly, the chlorophyll content declined throughout in these mutant lines (Figure 3C). The similar expression pattern of the genes for chlorophyll synthesis was found in the other green pod mutant lines (Supplementary Figure S3B). The genes involved in chlorophyll degradation were highly expressed in the M729Y with yellow pods; especially the expression levels of *RCCR* were high at the initial stage. However, the expression levels of genes for chlorophyll degradation in other green pod mutant lines were low (M729G, M693, and M756) (Figure 3D).

### Cellulose Contents and Transcriptional Abundance of Genes Involved in Cellulose Synthesis and Degradation

**FIGURE 4:**
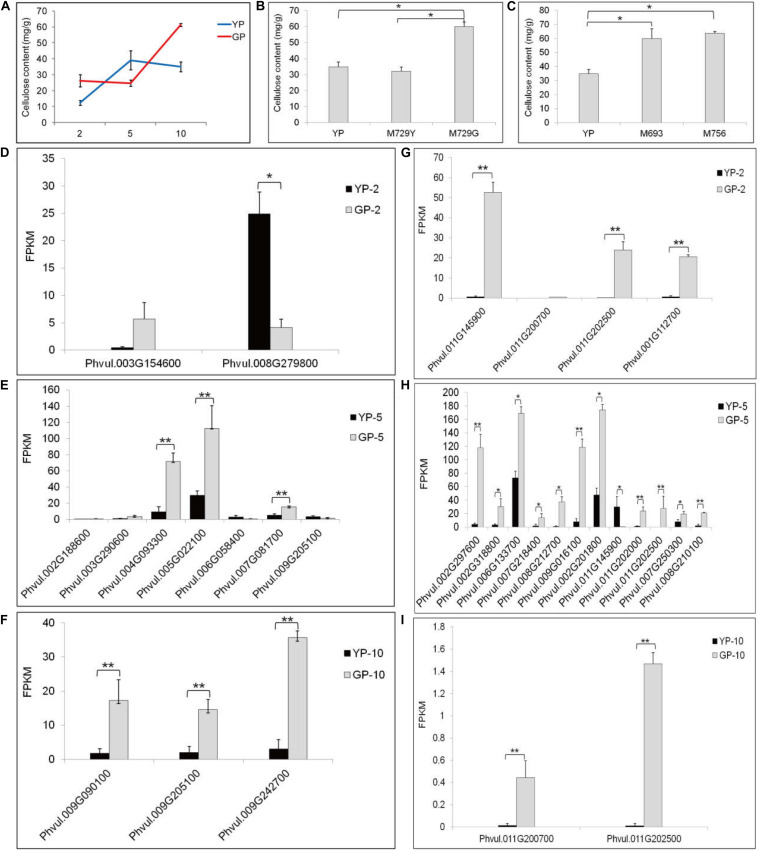
The cellulose content and the differential expression genes analysis in starch and sucrose metabolism pathway of YP and GP. **(A)** The cellulose content of wild type and green pod mutant. **(B,C)** The cellulose content in the other green pod mutant lines, **(B)** M729Y and M729G and **(C)** M693 and M756. **(D–F)** Differential expression genes for cellulose synthesis between YP-2 and GP-2, YP-5 and GP-5, and YP-10 and GP-10, respectively. *Phvul.004G093300* (cellulose synthase A catalytic subunit 2, *CESA2*), *Phvul.005G022100* (*CESA3*), *Phvul.002G188600*, *Phvul.009G090100* and *Phvul.009G242700* (*CESA4*), *Phvul.007G081700* (*CESA6*), *Phvul.003G154600*, *Phvul.009G205100* (*CESA7*), *Phvul.003G290600* (cellulose synthase-like protein G2, *CSLG2*), *Phvul.008G279800*, and *Phvul.006G058400* (cellulose synthase-like protein E6, *CSLE6*). **(G–I)** Differential expression genes for cellulose degradation between YP-2 and GP-2, YP-5 and GP-5, and YP-10 and GP-10, respectively. *Phvul.011G145900*, *Phvul.011G200700*, *Phvul.011G202500*, *Phvul.001G112700*, *Phvul.002G201800*, *Phvul.011G145900*, *Phvul.011G202000*, *Phvul.011G202500*, *Phvul.007G250300*, and *Phvul.008G210100* (beta-glucosidase). *Phvul.002G297600*, *Phvul.002G318800*, *Phvul.006G133700*, *Phvul.007G218400*, *Phvul.008G212700*, and *Phvul.009G016100* (endoglucanase). Error bars represent the standard deviation of three replicates. Data were analyzed using the *t*-test. *p < 0.05, **p < 0.01.

The tender pods of common bean are always consumed as vegetable, especially in Chinese recipes. In some cultivars, fibrosis in the pod leads to a hardened pod and loss of flavor. Therefore, eliminating fibrosis is a main target for breeding. After measuring, the cellulose content in the green pod mutant was higher than that in the wild type (Figure 4A). This result was also verified in other green pod mutant lines; the green pod mutant lines had higher cellulose content (Figures 4B,C). Therefore, we focused on cellulose synthesis and degradation and cellulose synthase genes. The genes of *Phvul.004G093300*, *Phvul.005G022100*, and *Phvul.009G242700* participating in cellulose synthesis had distinctly higher expression in GP than in YP samples (Figures 4D–F). The cellulose in the green pod mutant appeared to rapidly synthesized, leading to a higher cellulose content. There were also more genes involved in cellulose degradation in GP than in YP, which might be due to the degradation activity of cellulose being triggered by a higher cellulose content (Figures 4G–I).

### Real-Time Quantitative PCR Validation

**FIGURE 5:**
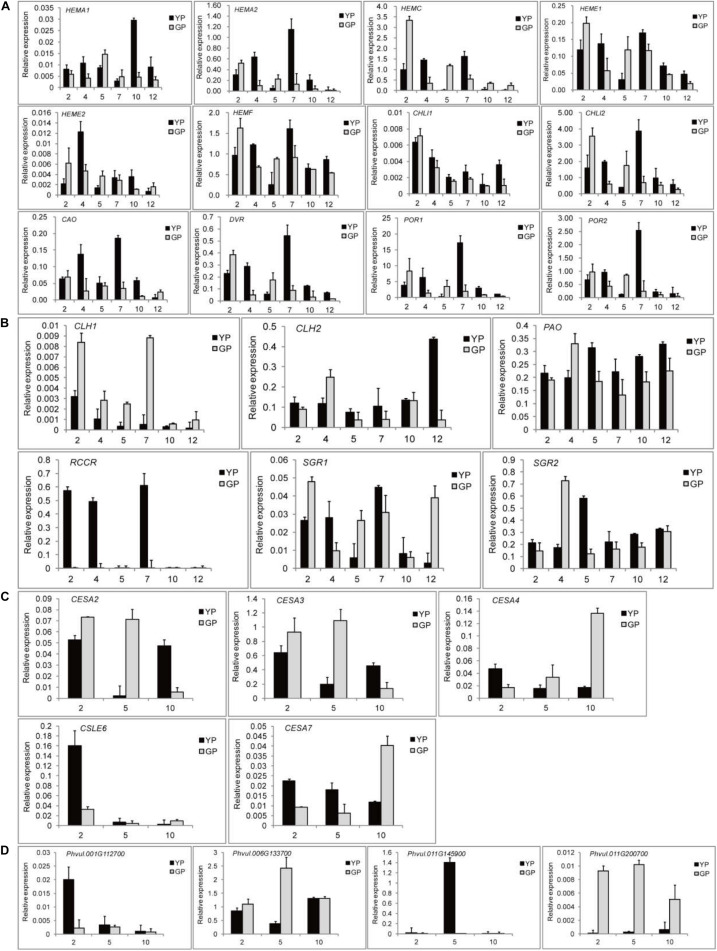
Quantitative real-time PCR (RT-qPCR) validation of DEGs found throughout transcriptome between the wild type and mutant. **(A)** DEGs involved in chlorophyll synthesis. **(B)** DEGs involved in chlorophyll degradation. **(C)** DEGs involved in cellulose synthesis. **(D)** DEGs involved in cellulose degradation.

To validate the key DEGs, the DEGs that showed significant differences in expression levels were selected: 12 chlorophyll synthesis pathway genes, 6 chlorophyll degradation genes, and 5 and 4 genes for cellulose synthesis and degradation, respectively. To investigate the changes in expression levels of the genes related to chlorophyll pathway, six different development stages of pods were selected. The results of RNA-seq were confirmed by RT-qPCR. Particularly, some genes, such as *HEMA2*, *HEME1*, *HEMF*, *CHLI2*, *CAO*, and *POR*, had a high expression when the pods were 7 cm (Figure 5A). For chlorophyll degradation, *PAO* and *SGR* played a significant role. The expression levels of *RCCR* in the green pod mutant were low; however, they were very high in the early stage in in the wild type (Figure 5B). The expression levels of cellulose synthesis and degradation genes showed a similar pattern to the transcriptome data (Figures 5C,D).

**FIGURE 6:**
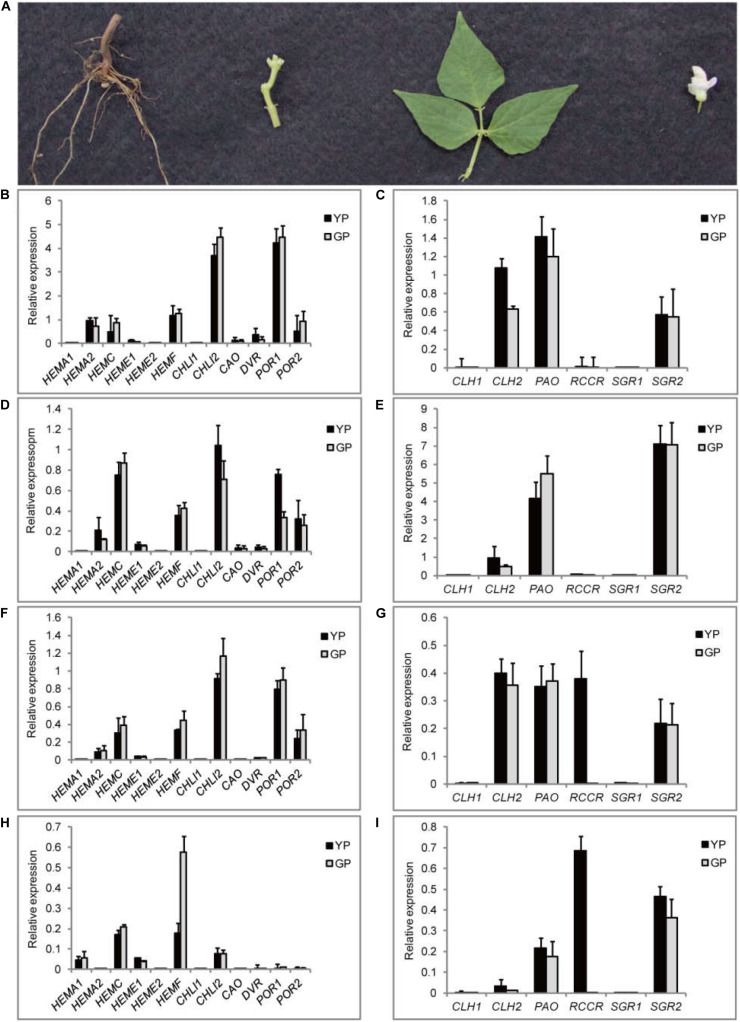
Transcription levels of 18 DEGs involving in chlorophyll synthesis and degradation pathway in different tissues. **(A)** The pictures of different organs of common bean. **(B–I)** The relative transcript levels of chlorophyll synthesis and degradation, respectively, in four different organs: **(B,C)** leaf, **(D,E)** flower, **(F,G)** stem, and **(H,I)** root.

**FIGURE 7:**
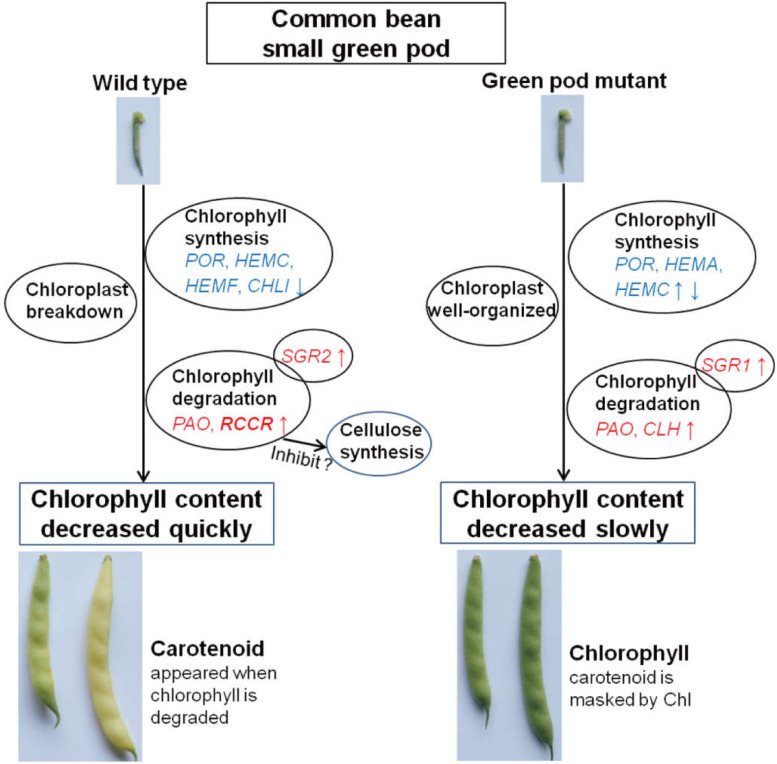
The proposed mechanism model of color conversion of the wild type and green pod mutant.

Meanwhile, the expression levels of the genes related to chlorophyll pathway were studied in different tissues (root, stem, leaf, and flower) using RT-qPCR (Figure 6). *CHLI2* and *POR* had a high expression in leaf, flower, and stem (Figures 6B,D,F); the expression of *HEMF* was higher in root (Figure 6H). *PAO* and *SGR2* had a high expression in leaf and flower (Figures 6C,E); the expression of *CLH2* was high in leaf and stem (Figures 6C,G). Compared to other genes, the expression levels of *CLH1*, *RCCR*, and *SGR1* were lower. Based on the above results, the mechanism model of pod degreening was speculated (Figure 7).

### Transcriptome and Metabolome Variations in the Flavonoid Pathway

**FIGURE 8:**
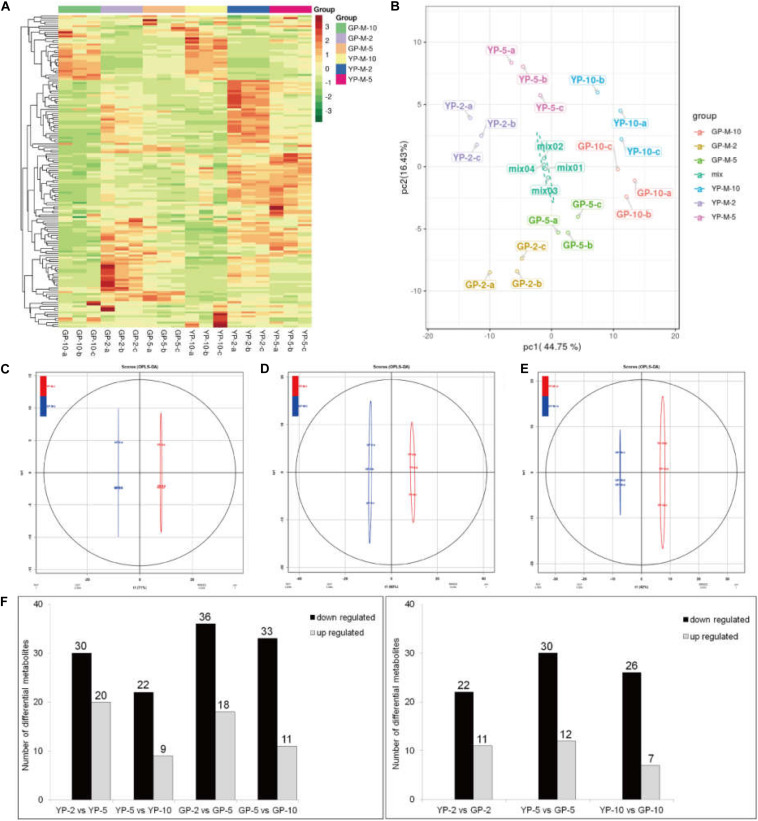
Heat map visualization, PCA, and OPLS-DA of the relative difference in flavonoids metabolites of YP and GP. **(A)** Heat map of all detected metabolites. The content of each metabolite was normalized to complete linkage hierarchical clustering. Each example is visualized in a single column, and each metabolite is represented by a single row. Red indicates high abundance; green showed low relative content. **(B)** Score plots for principle components 1 and 2 within and between groups. **(C–E)** OPLS-DA model plots for YP versus GP at different comparison groups in the same period. **(F)** The differentially metabolism analysis between wild type and green pod mutant at different comparable groups.

To determine the contents and composition variations of flavonoids during pod development, we performed metabolisms analysis by widely targeted metabolomics. A total of 154 flavonoid metabolites were identified from 18 samples (YP-2, YP-5, YP-10, GP-2, GP-5, and GP-10), each with three biological replicates. Setting VIP ≥ 1 together with fold change ≥ 2 or ≤ 0.5 as thresholds for significant differences, there were 33, 42, and 33 differential metabolites in the three comparison groups: YP-2 vs. GP-2, YP-5 vs. GP-5, and YP-10 vs. GP-10, respectively. Comparing the three comparison groups, 11, 12, and 7 metabolites were upregulated, and 22, 30, and 36 metabolites were downregulated (Figure 8).

Hierarchical cluster analysis of the flavonoids showed three main clusters based on the relative differences in accumulation patterns (Figure 8A), which indicated the complex differences in the metabolite levels. In the PCA plot, PCA on all metabolites indicated that PC1, explaining 44.75% of the total variance, separated samples of three different stages. PC2, accounting for 16.43% of the total variance, separated samples of YP from those of GP (Figure 8B). In the Orthogonal Projections to Latent Structures Discriminant Analysis (OPLS-DA) models, YP and GP samples were clearly separated (Figures 8C–E), indicating a major distinction in the metabolic profiles between the two different color groups.

In the comparison group, YP-2 vs. GP-2 (Table 1), naringin, vitexin 2″-*O*-beta-L-rhamnoside, and isotrifoliin were significantly higher in the YP-2, especially vitexin 2″-*O*-beta-L-rhamnoside (fold change = 0.0009, VIP = 3.26). Compared to YP-5 (Table 1), the content of three isoflavones (prunetin, biochanin A, and sissotrin) was higher in GP-5 and was 4. 15-, 2. 88-, and 8.81-fold, respectively. In YP-10, the content of 3,7-di-*O*-methylquercetin was significantly higher; other two flavonols (astragalin and kaempferin) were also higher in YP-10. Two flavonoids, luteolin and apigenin, were higher in GP-10. Compared to the wild type, isoflavones were largely accumulated in the green pod mutant, especially sissotrin. The content of five detected anthocyanin (oenin, pelargonin, cyanin, cyanidin, and keracyanin) was higher in the wild type at three different stages (Table 1).

**TABLE 1:** Differentially accumulated flavonoids compounds in the pod of wild type and green pod mutant.

YP-2 vs. GP-2	Index	Metabolite name	Class	Content		VIP	Fold change	Type
				YP-2	GP-2			
Flavonoid	pme0331	Naringin	Flavanone	79,200.00	38,166.67	1.03E + 00	4.82E – 01	Down
	pme3393	Fustin	Flavonol	18,166.67	43,833.33	1.16E + 00	2.41E + 00	Up
	pme0088	Luteolin	Flavone	219,333.33	593,666.67	1.16E + 00	2.71E + 00	Up
Flavone and flavonol	pme0363	Chrysoeriol	Flavone	265,333.33	633,333.33	1.09E + 00	2.39E + 00	Up
	pme3227	Vitexin 2″-*O*-beta-L-rhamnoside	Flavone	9,623.33	9.00	3.26E + 00	9.35E – 04	Down
	pme3212	Quercetin 3-*O*-glucoside	Flavonol	4,450,000.00	1,413,333.33	1.29E + 00	3.18E – 01	Down
Anthocyanin	pme0444	Malvidin 3-*O*-glucoside	Anthocyanins	196,333.33	90,933.33	1.04E + 00	4.63E – 01	Down
	pme1793	Pelargonin	Anthocyanins	330,666.67	89,533.33	1.38E +00	2.71E – 01	Down
	pme1777	Cyanidin 3,5-*O*-diglucoside	Anthocyanins	49,966.67	23,966.67	1.02E + 00	4.80E – 01	Down
YP-5 vs. GP-5				YP-5	GP-5			
Flavonoid	pme0199	Quercetin	Flavonol	488,000.00	179,666.67	1.03E +00	3.68E – 01	Down
	pme0088	Luteolin	Flavone	31,800.00	105,400.00	1.02E + 00	3.31E + 00	Up
	pme0196	Kaempferol	Flavonol	19,866.67	3,439.33	2.21E + 00	1.73E – 01	Down
Flavone and flavonol	pme3296	Kaempferin	Flavonol	12,933.33	3,746.67	1.20E + 00	2.90E – 01	Down
	pme1541	Acacetin	Flavone	9,696.67	38,300.00	1.22E + 00	3.95E + 00	Up
Isoflavonoid	pme3292	Prunetin	Isoflavone	11,070.00	45,933.33	1.27E + 00	4.15E + 00	Up
	pme3250	Biochanin A	Isoflavone	849.33	6,273.33	2.07E + 00	7.39E + 00	Up
	pme3399	Sissotrin	Isoflavone	10,406.67	91,633.33	1.59E + 00	8.81E + 00	Up
Anthocyanin	pme3609	Cyanidin	Anthocyanins	160,000.00	64,733.33	1.02E + 00	4.05E – 01	Down
YP-10 vs. GP-10				YP-10	GP-10			
Flavonoid	pme0088	Luteolin	Flavone	3,170.00	7,193.33	1.05E +00	2.27E +00	Up
	pme0379	Apigenin	Flavone	4,439.33	10,163.33	2.39E + 00	2.29E + 00	Up
Flavone and flavonol	pme3290	3,7-Di-*O*-methylquercetin	Flavonol	639.33	9.00	1.16E + 00	1.41E – 02	Down
	pmb0604	Astragalin	Flavonol	4,780,000.00	1,863,333.33	1.29E +00	3.90E – 01	Down
	pme3296	Kaempferin	Flavonol	4,206.67	1,476.67	1.18E + 00	3.51E – 01	Down
Isoflavonoid	pme3502	Ononin	Isoflavone	27,300.00	10,116.67	1.30E + 00	3.71E – 01	Down
	pme3399	Sissotrin	Isoflavone	9.00	956.00	1.24E + 00	1.06E + 02	Up
Anthocyanin	pme1773	Cyanidin 3-*O*-rutinoside	Anthocyanins	327,333.33	134,266.67	1.11E + 00	4.10E – 01	Down

Analysis of transcriptome data revealed that most DEGs involved in the flavonoids pathway showed to be downregulated (Supplementary Figures S4A,B); there were more DEGs in flavones and isoflavones pathways in the wild type. Many genes of YP and GP samples showed similar expression trends, such as *DFR2*, *ANR*, *LAR*, *DFR3*, *IF7MAT6*, *VR4* (Supplementary Figures S4A–D). There were more genes expressed in the pods at 5 cm long (YP-5, GP-5) (Supplementary Figure S5). By KEGG enrichment analysis, the DEGs (YP-5/GP-5) of isoflavonoid biosynthesis pathway were enriched. The genes involving in anthocyanin synthesis were rarely detected.

## Discussion

### The Molecular Mechanism of Degreening Process

The loss of green color in senescent leaves and ripening fruits is a noticeable natural phenomenon. It is well known that the color conversion is generally accompanied by chlorophyll degradation; the chlorophyll content constantly decreased during the green-peel fruit ripening (Gambi et al., 2018). During the pod development of the golden hook, the green pod color rapidly turned yellow, showing an inherent characteristic as an ecotype of the common bean. The yellow pods of the golden hook are tender with less cellulose and easy to cook.

In this study, the color transition from green to yellow resulted from the rapid degradation of chlorophyll and the appearance of carotenoids. The *PAO* degradation pathway of chlorophyll is a multistep enzymatic process, in which *CLH*, *PAO*, *RCCR*, and stay-green (*SGR*) genes have been documented (Tsuchiya et al., 1999; Pruzinska et al., 2003; Harpaz-Saad et al., 2007). *CLH*, the first step enzyme in the chlorophyll catabolic pathway, acts as a rate-limiting enzyme controlled via posttranslational regulation (Harpaz-Saad et al., 2007). In this study, *CLH1* showed low expression in both YP and GP, whereas *CLH2* was upregulated in the mutant during pod development. Previous researches had shown that *PAO* and *RCCR* participated in the chlorophyll catabolic pathway. *PAO* is a key enzyme and is upregulated during leaf senescence (Ginsburg et al., 1994; Hörtensteiner et al., 1995; Pruzinska et al., 2003). The *PAO* activity seemed to be responsible for the stay-green genotype in pepper (Roca and Minguez-Mosquera, 2006). The expression of *RCCR* is constitutive (Rodoni et al., 1997; Tsuchiya et al., 1999). In this study, the expression levels of *PAO* were high in both wild type and green pod mutant but particularly were increased in the wild type during pod development. The expression of *RCCR* was very high in small tender pod of the wild type and then declined; this means that *RCCR* acted as key enzyme for chlorophyll degradation in early stages in the wild type. There was an extremely low expression of *RCCR* in the green pod mutant lines. Some studies suggested that *SGR* may take part in the chloroplast breakdown process (Kusaba et al., 2007; Park et al., 2007). *SGR*, as a chloroplast protein, has an important role in chlorophyll degradation by catabolic enzymes and proteases through inducing LHCPII disassembly (Aubry et al., 2008). Our results support this notion; the expression levels of *SGR2* showed significant changes, but the expression levels of *SGR1* were low. The phytohormone ethylene and jasmonic acid could affect the chlorophyll degradation pathway; they could increase the expression of Chl catabolic genes (Fang et al., 2020; Lv et al., 2020). Compared to the green pod mutant, the expression levels of chlorophyll degradation genes in the wild type was earlier and higher. *PAO*, *RCCR*, and *SGR* might be the key genes in the golden hook involved in chlorophyll degradation; especially *RCCR* played a much more important role. Analysis of the resequencing data of the wild type and green pod mutant revealed that there were some no-sense mutations occurring in the genes involving in chlorophyll degradation (Supplementary Table S3).

In this study, comparing wild type to green pod mutant, only the pods turned yellow during pod development; there were no noticeable color differences in the other tissues. The expression levels of the genes for chlorophyll degradation were low in the leaf and stem. According to the results of this study, we developed a mechanic model describing degreening process (Figure 7), in which the chlorophyll content decreased, and the structure of chloroplast breakdown in the wild type during pod development. The expression levels of chlorophyll degradation genes were higher and earlier in the pods of the wild type leading to rapid degreening, especially *RCCR* showed a significantly differential expression between the wild type and green pod mutant.

### The Internal Mechanism of Phenotypic Changes in Cellulose Content

A common feature for different wall types of plants is the prevalence of cellulose, which consists of β-1,4 glucan chains that are synthesized by *CESA* protein complexes (CSCs). The CSC typically consists of different heterotrimeric *CESA* configurations. Different *CESA* has respective roles; during primary wall synthesis, the CSC contains *CESA1*, *3* and one *CESA6*-like subunit (Desprez et al., 2007; Persson et al., 2007). By contrast, the secondary wall-synthesizing CSCs contain *CESA4*, *7*, and *8* (Taylor et al., 2003). In this study, some *CESA* showed differential expression. The genes (*Phvul.004G093300*, *Phvul.005G022100*, and *Phvul.009G242700*) participating in cellulose synthesis had distinct higher expression in GP than in YP samples. There were also more genes involving in cellulose degradation in GP; the cellulose in the green pod mutant showed rapid synthesis. The high cellulose content in pods might be one of the reasons that the green pod mutant was much harder.

Cellulose biosynthesis is a complex biochemical process, which cannot be reproduced *in vitro*. Fiber content is a key index of common bean quality; the common bean with low fiber content has a good taste. Golden hook is a very popular ecotype of the common bean and has a very low cellulose content in fresh pods. There were also some genes for cellulose synthesis having relative low expression in the wild type. Conversely, there was relatively high cellulose content in green pod mutants at the same stage; MeJA treatment promoted peach fruit chlorophyll degradation and affected fruit softening through increasing the expression of cellulase (Wei et al., 2017). The genes for cellulose synthesis pathway might be downregulated by the genes involved in degreening of pods.

Sucrose is the main form of assimilated carbon that is produced during plant photosynthesis; UDP-glucose can be transformed from sucrose catalyzed by *SuSy*. UDP-glucose was just the immediate substrate for cellulose synthesis (Nakai et al., 1999; Verbancic et al., 2018). It was speculated that photosynthesis of green pod mutants might provide synthetic substrate for cellulose, but the intrinsic interaction need to be further studied.

### Transcriptome and Metabolome Analyses Involved in Flavonoid Pathway

Flavonoids, important secondary metabolites found in plants, contribute to plant environmental adaptation, fruit development (Petroni and Tonelli, 2011; Li et al., 2019), and human health (Alipour et al., 2016). These compounds are accumulated in various tissues and are also the direct factors that cause color variations in many fruits or flowers. Combined metabolome and transcriptome analyses were carried out with *Ficus carica* L.; cyaniding *O*-malonylhexoside demonstrated a 3,992-fold increase; cyaniding 3-*O*-gglucoside, cyanidin-3, and 5-*O*-diglucoside were upregulated 100-fold, revealing the anthocyanins underlying the purple mutation (Wang et al., 2017). The pod color and pattern of the common bean is colorful; some varieties having red or purple patterns could be rich in anthocyanin. On the other hand, the common bean is a special legume variety that is abundant in flavonoids, especially isoflavones. In a purple kidney bean cultivar, malvidin 3,5-diglucoside was identified as the major anthocyanin in the pod skin by HPLC-ESI-MS (Hu et al., 2015). Naringin, vitexin 2″-*O*-beta-L-rhamnoside, and isotrifoliin demonstrated significantly higher contents in the YP-2. Compared to YP-5, the content of three isoflavonoid (prunetin, biochanin A, and sissotrin) was higher in GP-5. Only five anthocyanin were detected (oenin, pelargonin, cyanin, cyanidin, and keracyanin), and all these five anthocyanins were significantly higher (VIP >1, fold change <0.5) in the wild type than that in the green pod mutant. When the pod length reached to 10 cm, cyanidin 3-*O*-rutinoside (keracyanin) was the sole anthocyanin with datable difference between GP-10 and YP-10. Thus, it could be speculated that anthocyanins content could be influenced by the degreening to certain extent but not significantly.

### The Whole Nutrition Value and Breeding Strategy in Common Bean

The common bean is a nutrient-rich food that contains nutrients essential for humans, such as proteins, minerals, vitamins, carbohydrates, and fiber. The protein quality in the common bean is high, and many cultivars have sufficiently high levels of essential and non-essential amino acids to meet daily nutritional needs (Ribeiro et al., 2007). Although the protein content of the common bean is approximately 50% of that of soybean, the digestibility of protein is higher (78.70%). In view of the high mineral content, common beans benefit health and can be used to cure a number of mineral deficiencies (Mesquita et al., 2007).

The yield potential of the common bean is determined by growth habit, pod number, maturity features, and seed characteristics. In addition to the yield, the external phenotype, taste, cook characteristic, and disease and abiotic stress tolerance are prime goals in breeding. As a high-quality variety of oil beans, Dalong 1 has high nutritional value, less cellulose, and good taste. In this study, we studied the color conversion of pods and flavonoids metabolism between the wild type and green pod mutants; it was helpful to understand the metabolic process of nutrients and provide a theoretical basis for breeding.

## Data Availability Statement

All the information regarding our sequencing data we deposited at National Genomics Data Center BIG Sub repository: Project No: PRJCA002296, GSAs: CRA002791, Experiment acc: CRX119315–CRX119332, Samples acc: SAMC138882–SAMC1388.

## Author Contributions

BH designed the experiments, carried out the research, and wrote and revised the manuscript. JY, DZ, HW, and NG prepared plant materials. JZ and YG helped to collected samples in the field. KX, HZ, and ZX gave important suggestions and assisted in data analysis. All authors participated in this study and approved the final version of the manuscript.

## Conflict of Interest

The authors declare that the research was conducted in the absence of any commercial or financial relationships that could be construed as a potential conflict of interest.

## Footnotes

1 http://plants.ensembl.org/index.html

2 www.anpel.com.cn/cnw

3 http://www.anpel.com.cn/

4 www.shimadzu.com.cn/

5 www.appliedbiosystems.com.cn/

## Supplementary Material

FIGURE S1 The phenotype of pods of the wild type and green pod mutant.

FIGURE S2 KEGG and GO enrichment analysis in wild type and green pod mutant.

FIGURE S3 Heat map diagram of expression levels of the genes for chlorophyll synthesis in YP, GP and other green pod mutant lines.

FIGURE S4 The heat map of DEGs in flavones and isoflavones pathway.

FIGURE S5 The differentially expression genes analysis in flavonoids metabolism pathway between YP and GP.

TABLE S1 Statistics for the number of clean data.

TABLE S2 List of genes and primers for RT-qPCR.

TABLE S3 Statistics of SNP variants of the genes for chlorophyll degradation.

## Abbreviations

1-MCP: 1-methylcyclopropene
*ANR*: anthocyanidin reductase
*CESA*: cellulose synthase A
*CHLI*: Mg chelatase I subunit
*CHS*: chalcone synthase
*CLH*: chlorophyllase
DEGs: differential expression genes
*DFR*: dihydroflavonol reductase
GO: Gene Ontology
GP: the green pod mutant M628
HCA: hierarchical cluster analysis
*HEMA*: glutamyl tRNA reductase
*HEMC*: hydroxymethylbilane synthase
HPLC: high-performance liquid chromatography
*IF7MAT6*: isoflavone 7-*O*-glucosyltransferase
KEGG: Kyoto Encyclopedia of Genes and Genomes
*Kor*: cellulase
*LAR*: leucoanthocyanidin reductase
M693, M756: two green pod mutant lines
M729G: one mutant line with green pods
M729Y: one mutant line with yellow pods
*NOL*: NYC1-like
*NYC1*-like: non-yellow coloring 1-like
*PAO*: pheophorbide a oxygenase
PCA: principal component analysis
*POR*: protochlorophyllide oxidoreductase
QTL: quantitative trait loci
*RCCR*: red chlorophyll catabolite reductase
RT-qPCR: real-time quantitative PCR
*SGR1*: stay green 1
*SuSy*: sucrose synthase
UDP-glucose: uridine diphosphate glucose
*VR4*: vestitone reductase
YP: wild type Dalong 1.

## References

[B1] Alipour B. Rashidkhani B. Edalati S. (2016). Dietary flavonoid intake, total antioxidant capacity and lipid oxidative damage: a cross-sectional study of Iranian women. *Nutrition* 32 566–572. 10.1016/j.nut.2015.11.011 26830011

[B2] Aubry S. Mani J. Hortensteiner S. (2008). Stay-green protein, defective in Mendel’s green cotyledon mutant, acts independent and upstream of pheophorbide a oxygenase in the chlorophyll catabolic pathway. *Plant Moecularl Biol.* 67 243–256. 10.1007/s11103-008-9314-8 18301989

[B3] Borges A. Tsai S. M. Caldas D. G. (2012). Validation of reference genes for RT-qPCR normalization in common bean during biotic and abiotic stresses. *Plant Cell Rep.* 31 827–838. 10.1007/s00299-011-1204-x 22193338

[B4] Charoenchongsuk N. Ikeda K. Itai A. Oikawa A. Murayama H. (2015). Comparison of the expression of chlorophyll-degradation-related genes during ripening between stay-green and yellow-pear cultivars. *Sci. Hortic.* 181 89–94. 10.1016/j.scienta.2014.10.005

[B5] Cheng Y. Dong Y. Yan H. Ge W. Shen C. Guan J. (2012). Effects of 1-MCP on chlorophyll degradation pathway-associated genes expression and chloroplast ultrastructure during the peel yellowing of Chinese pear fruits in storage. *Food Chem.* 135 415–422. 10.1016/j.foodchem.2012.05.017 22868108

[B6] Coleman H. D. Yan J. Mansfield S. D. (2009). Sucrose synthase affects carbon partitioning to increase cellulose production and altered cell wall ultrastructure. *PNAS* 106 13118–13123. 10.1073/pnas.0900188106 19625620 PMC2722352

[B7] Cui B. L. Hu Z. L. Zhang Y. J. Hu J. Y. Yin W. C. Feng Y. (2016). Anthocyanins and flavonols are responsible for purple color of *Lablab purpureus* (L.) sweet pods. *Plant Physiol. Biochem.* 103 183–190. 10.1016/j.plaphy.2016.03.011 26995313

[B8] Desprez T. Juraniec M. Crowell E. F. Jouy H. Pochylova Z. Parcy F. (2007). Organization of cellulose synthase complexes involved in primary cell wall synthesis in Arabidopsis thaliana. *PNAS* 104 15572–15577. 10.1073/pnas.0706569104 17878303 PMC2000492

[B9] Fang H. X. Luo F. Li P. X. Zhou Q. Zhou X. Wei B. D. (2020). Potential of jasmonic acid (JA) in accelerating postharvest yellowing of broccoli by promoting its chlorophyll degradation. *Food Chem.* 309 1–11. 10.2212/spr.2008.6.8 31780227

[B10] Gambi F. Pilkington S. M. McAtee P. A. Donati I. Schaffer R. J. Montefiori M. (2018). Fruit of three kiwifruit (*Actinidia chinensis*) cultivars differ in their degreening response to temperature after harvest. *Postharvest Biol. Technol.* 141 16–23. 10.1016/j.postharvbio.2018.03.009

[B11] Ginsburg S. Schellenberg M. Matile P. (1994). Cleavage of chlorophyll-Porphyrin. *Plant Physiol.* 105 545–554. 10.1104/pp.105.2.545 12232222 PMC159392

[B12] Harpaz-Saad S. Azoulay T. Arazi T. Ben-Yaakov E. Mett A. Shiboleth Y. M. (2007). Chlorophyllase is a rate-limiting enzyme in chlorophyll catabolism and is posttranslationally regulated. *Plant Cell.* 19 1007–1022. 10.1105/tpc.107.050633 17369368 PMC1867358

[B13] Hörtensteiner S. Vicentini F. Matile P. (1995). Chlorophyll breakdown in senescent cotyledons of rape, *Brassica napus* L. : enzymatic cleavage of phaeophorbide a in vitro. *New Phytologist.* 129 237–246. 10.1111/j.1469-8137.1995.tb04293.x 33874553

[B14] Hu J. T. Chen G. P. Zhang Y. Y. Cui B. L. Yin W. C. Yu X. H. (2015). Anthocyanin composition and expression analysis of anthocyanin biosynthetic genes in kidney bean pod. *Plant Physiol. Biochem.* 97 304–312. 10.1016/j.plaphy.2015.10.019 26512970

[B15] Kanehisa M. Araki M. Goto S. Hattori M. Hirakawa M. Itoh M. (2008). KEGG for linking genomes to life and the environment. *Nucleic Acids Res.* 36 480–484. 10.1093/nar/gkm882 18077471 PMC2238879

[B16] Kovinich N. Kayanja G. Chanoca A. Riedl K. Otegui M. S. Grotewold E. (2014). Not all anthocynins are born equal: distinct patterns induced by stress in *Arabidopsis*. *Planta* 240 671–687. 10.1007/s00425-014-2079-1 PMC4200348 24903357

[B17] Kusaba M. Ito H. Morita R. Iida S. Sato Y. Fujimoto M. (2007). Rice NON-YELLOW COLORING1 is involved in light-harvesting complex II and grana degradation during leaf senescence. *Plant Cell* 19 1362–1375. 10.1105/tpc.106.042911 17416733 PMC1913755

[B18] Li B. Dewey C. N. (2011). RSEM: accurate transcript quantification from RNA-Seq data with or without a reference genome. *BMC Bioinformatics* 12:323. 10.1186/1471-2105-12-323 21816040 PMC3163565

[B19] Li H. Y. Lv Q. Y. Ma C. Qu J. T. Cai F. Deng J. (2019). Metabolite profiling and transcriptome analyses provide insights into the flavonoid biosynthesis in the developing seed of Tartary Buckwheat (*Fagopyrum tataricum*). *J. Agric/. Food Chem.* 67 11262–11276. 10.1021/acs.jafc.9b03135 31509416

[B20] Li Y. Yang K. Yang W. Chu L. W. Chen C. H. Zhao B. (2017). Identification of QTL and qualitative trait loci for agronomic traits using SNP markers in the adzuki bean. *Front. Plant Sci.* 8:840. 10.3389/fpls.2017.00840 28580006 PMC5437206

[B21] Lv J. Y. Zhang M. Y. Bai L. Han X. Z. Ge Y. H. Wang W. H. (2020). Effects of 1-methylcyclopropene (1-MCP) on the expression of genes involved in the chlorophyll degradation pathway of apple fruit during storage. *Food Chem.* 308 1–7. 10.1016/j.foodchem.2019.125707 31669943

[B22] Mesquita F. R. Corrêa A. D. Abreu C. M. Lima R. A. Abreu A. F. (2007). Linhagens de feijão (*Phaseolus vulgaris* L.): composição química e digestibilidade proteica. *Ciência e Agrotecnol.* 31 1114–1121. 10.1590/s1413-70542007000400026

[B23] Motamayor J. C. Mockaitis K. Schmutz J. Haiminen N. Livingstone D. Cornejo O. (2013). The genome sequence of the most widely cultivated cacao type and its use to identify candidate genes regulating pod color. *Genome Biol.* 14:r53. 10.1186/gb-2013-14-6-r53 PMC4053823 23731509

[B24] Nakai T. Tonouchi N. Konishi T. Kojima Y. Tsuchida T. Yoshinaga F. (1999). Enhancement of cellulose production by expression of sucrose synthase in *Acetobacter xylinum*. *PNAS* 96 14–18. 10.1073/pnas.96.1.14 9874763 PMC15084

[B25] Okonkwo C. A. Clayberg C. D. (1984). Genetics of flower and pod color in *Phaseolus vulgaris*. *J. Heredity.* 75 440–444. 10.1093/oxfordjournals.jhered.a109981

[B26] Park S. Y. Yu J. W. Park J. S. Li J. J. Yoo S. C. Lee N. Y. (2007). The senescence-induced staygreen protein regulates chlorophyll degradation. *Plant Cell.* 19 1649–1664. 10.1105/tpc.106.044891 17513504 PMC1913741

[B27] Persson S. Paredez A. Carroll A. Palsdottir H. Doblin M. Poindexter P. (2007). Genetic evidence for three unique components in primary cell-wall cellulose synthase complexes in Arabidopsis. *PNAS* 104 15566–15571. 10.1073/pnas.0706592104 17878302 PMC2000526

[B28] Petroni K. Tonelli C. (2011). Recent advances on the regulation of anthocyanin synthesis in reproductive organs. *Plant Sci.* 181 219–229. 10.1016/j.plantsci.2011.05.009 21763532

[B29] Pruzinska A. Tanner G. Anders I. Roca M. Hortensteiner S. (2003). Chlorophyll breakdown: pheophorbide a oxygenase is a Rieske-type iron-sulfur protein, encoded by the accelerated cell death 1 gene. *PNAS* 100 15259–15264. 10.1073/pnas.2036571100 14657372 PMC299977

[B30] Ribeiro N. D. Londero P. M. Filho A. C. Jost E. Poersch N. L. Mallmann C. A. (2007). Composição de aminoácidos de cultivares de feijão e aplicações para o melhoramento genético. *Pesquisa Agropecuária Brasil.* 42 1393–1399.

[B31] Roca M. Minguez-Mosquera M. I. (2006). Chlorophyll catabolism pathway in fruits of *Capsicum annuum* (L.): stay-green versus red fruits. *J. Agric. Food Chem.* 54 4035–4040. 10.1021/jf060213t 16719531

[B32] Rodoni S. Mühlecker W. Anderl M. Krautler B. Moser D. Thomas H. (1997). Chlorophyll breakdown in senescent chloroplasts. *Plant Physiol.* 115 669–676. 10.1104/pp.115.2.669 12223835 PMC158527

[B33] Sakuraba Y. Park S. Y. Kim Y. S. Wang S. H. Yoo S. C. Hortensteiner S. (2014). *Arabidopsis* STAY-GREEN2 is a negative regulator of chlorophyll degradation during leaf senescence. *Mol. Plant* 7 1288–1302. 10.1093/mp/ssu045 24719469

[B34] Tanaka Y. Sasaki N. Ohmiya A. (2008). Biosynthesis of plant pigments: anthocyanins, betalains and carotenoids. *Plant J.* 54 733–749. 10.1111/j.1365-313x.2008.03447.x 18476875

[B35] Taylor N. G. Howells R. M. Huttly A. K. Vickers K. Turner S. R. (2003). Interactions among three distinct CesA proteins essential for cellulose synthesis. *PNAS* 100 1450–1455. 10.1073/pnas.0337628100 12538856 PMC298793

[B36] Tharanathan R. N. Mahadevamma S. (2003). Grain legumes- a boon to human nutrition. *Trends Food Sci. Technol.* 14 507–518. 10.1016/j.tifs.2003.07.002

[B37] Tsuchiya T. Ohta H. Okawa K. Iwamatsu A. Shimada H. Masuda T. (1999). Cloning of chlorophyllase, the key enzyme in chlorophyll degradation: finding of a lipase motif and the induction by methyl jasmonate. *PNAS* 92 15362–15367. 10.1073/pnas.96.26.15362 10611389 PMC24824

[B38] Verbancic J. Lunn J. E. Stitt M. Persson S. (2018). Carbon supply and the regulation of cell wall synthesis. *Mol. Plant* 11 75–94. 10.1016/j.molp.2017.10.004 29054565

[B39] Wang H. Zhang H. Yang Y. Li M. F. Zhang Y. T. Liu J. S. (2019). The control of red color by a family of MYB transcription factors in octoploid strawberry (Fragaria x ananassa) fruits. *Plant Biotechnol. J.* 18 1169–1184. 10.1111/pbi.13282 31647169 PMC7152614

[B40] Wang Z. Cui Y. Vainstein A. Chen S. Ma H. (2017). Regulation of Fig (*Ficus carica* L.) fruit color: metabolomic and transcriptomic analyses of the flavonoid biosynthetic pathway. *Front. Plant Sci.* 8:1990. 10.3389/fpls.2017.01990 29209349 PMC5701927

[B41] Wei J. Wen X. Tang L. (2017). Effect of methyl jasmonic acid on peach fruit ripening progress. *Sci. Hortic.* 220 206–213. 10.1016/j.scienta.2017.03.004

[B42] Xu P. Su H. Jin R. Mao Y. X. Xu A. Cheng H. Y. (2020). Shading effects on leaf color conversion and biosynthesis of the major secondary metabolites in the Albina Tea cultivar “Yujinxiang”. *J. Agric. Food Chem.* 68 2528–2538. 10.1021/acs.jafc.9b08212 32011878

[B43] Yuste-Lisbona F. J. Gonzalez A. M. Capel C. Garcia-Alcazar M. Capel J. Ron A. M. (2014). Genetic varation underlying pod size and color traits of common bean depends on quantitative trait loci with epistatic effects. *Mol. Breed.* 33 939–952. 10.1007/s11032-013-0008-9

[B44] Zhuang H. M. Lou Q. Liu H. F. Han H. W. Wang Q. Tang Z. H. (2019). Differential regulation of anthocyanins in green and purple turnips revealed by combined de novo transcriptome and metabolome analysis. *Int. J. Mol. Sci.* 20 1–18. 10.3390/ijms20184387 PMC6769466 31500111

