# Supplementary material for: Enhanced Chlorophyll Degradation Triggers the Pod Degreening of “Golden Hook,” a Special Ecotype in Common Bean (Phaseolus vulgaris L.)
Source: Front Genet. 2020 Oct 6;11:570816. doi: 10.3389/fgene.2020.570816 (PMC7573562; doi:10.3389/fgene.2020.570816)
Supplement: FIGURE S1 — The phenotype of pods of the wild type and green pod mutant. [file Data_Sheet_1.docx]

Figure S1. The phenotype of pods of the wild type and green pod mutant. (A) The phenotypic picture of the whole plant in the field, the left was the wild type; the right was green pod mutant. Scale bar: 5 cm. (C, D) Pod and seed size of the wild type and green pod mutant, respectively.

Figure S2. KEGG and GO enrichment analysis in wild type and green pod mutant. (A) Top 20 significantly enriched KEGG pathway. (B) Top 20 significantly enriched GO pathway.

Figure S3. Heat map diagram of expression abundance of the genes for chlorophyll synthesis in YP, GP and other green pod mutant lines. (A) The expression abundance of chlorophyll synthesis genes in YP and GP. (B) The expression abundance of chlorophyll synthesis genes in other green pod mutant lines (M729Y, M729G, M693 and M756).

Figure S4. The heat map of DEGs in flavones and isoflavones pathway. (A, B) Heat map of DEGs in flavones pathway of the wild type (YP, A) and green pod mutant (GP, B), respectively. (C, D) Heat map of DEGs in isoflavones pathway of the wild type (YP, C) and green pod mutant (GP, D), respectively.

Figure S5. The differentially expression genes analysis in flavonoids metabolism pathway between YP and GP. (A) The DEGs in flavonoids biosynthesis between YP-2 and GP-2, YP-5 and GP-5, YP-10 and GP-10. (B) The DEGs in isoflavonoid biosynthesis between YP-2 and GP-2, YP-5 and GP-5. (C) The DEGs in anthocyanin biosynthesis between YP-5 and GP-5.


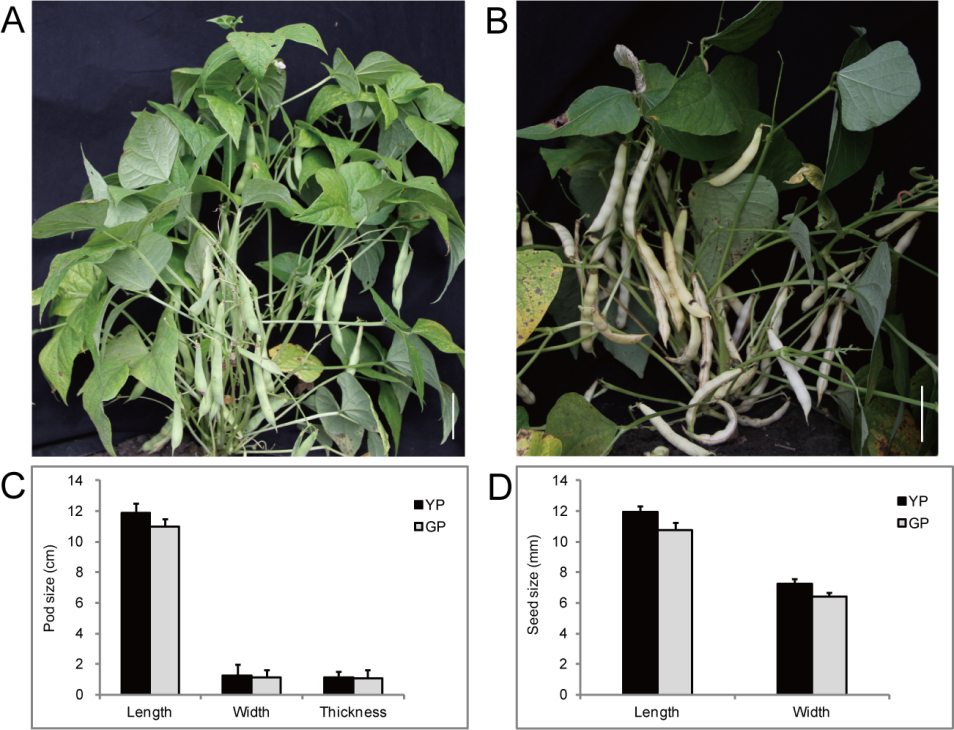


Figure S1. The phenotype of pods of the wild type and green pod mutant.


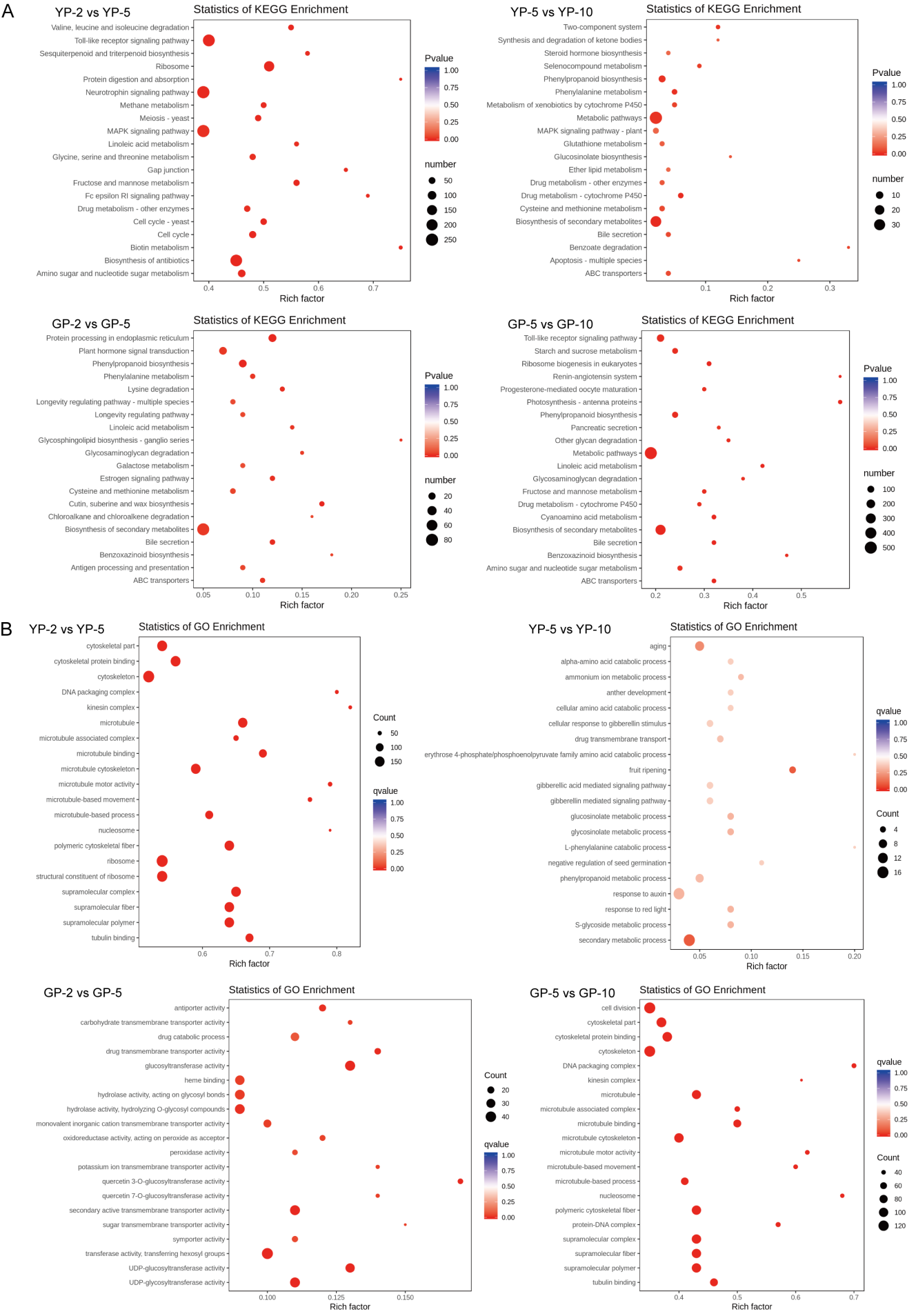


Figure S2. KEGG and GO enrichment analysis in wild type and green pod mutant.


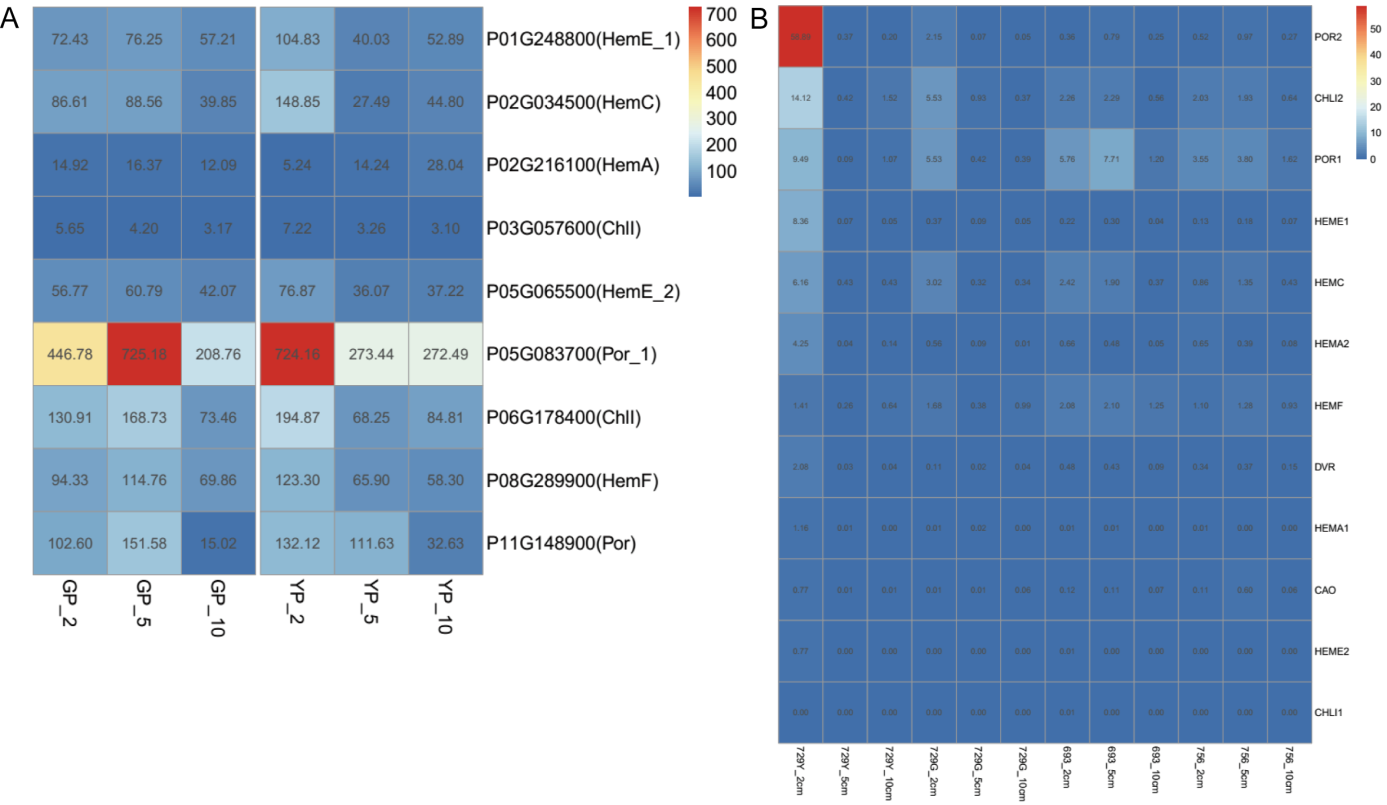


Figure S3. Heat map diagram of expression abundance of the genes for chlorophyll synthesis in YP, GP and other green pod mutant lines.


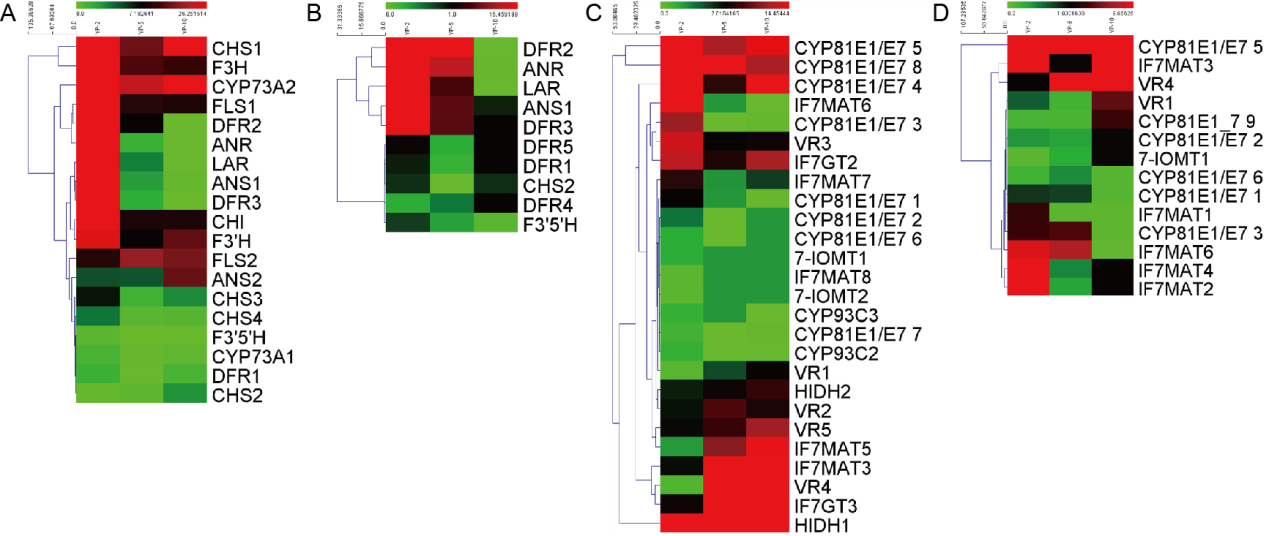


Figure S4. The heat map of DEGs in flavones and isoflavones pathway.


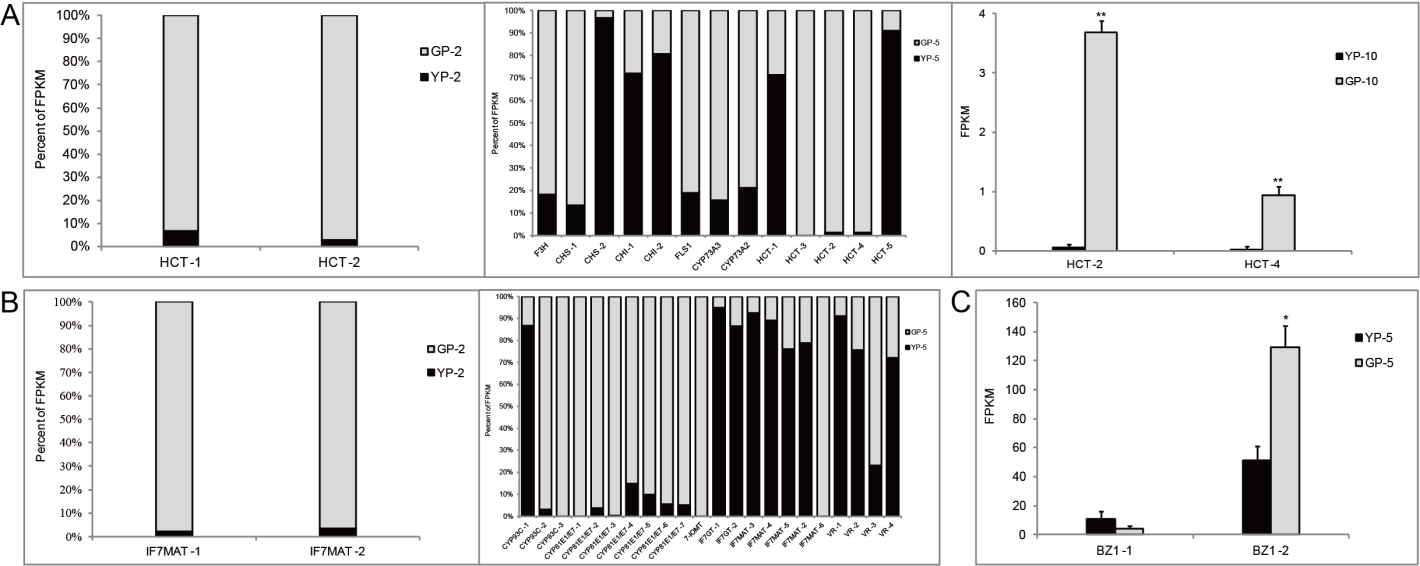


Figure 5. The differentially expression genes analysis in flavonoids metabolism pathway between YP and GP.
